# Bleeding on Probing as a Predictor of Peri‐Implant Bone Loss During Supportive Care: A Prospective Cohort Study

**DOI:** 10.1111/cid.70138

**Published:** 2026-03-24

**Authors:** Beatriz de Tapia, Gloria Torrens, Francesco di Leone, Ettore Amerio, Cristina Valles, Jose Nart, Alberto Monje

**Affiliations:** ^1^ Department of Periodontics Universitat Internacional de Catalunya Barcelona Spain; ^2^ Department of Biomedical, Surgical and Dental Sciences, Unit of Periodontology University of Milan Milan Italy; ^3^ Fondazione IRCCS Ca' Granda Polyclinic Milan Italy; ^4^ Department of Periodontics and Oral Medicine University of Michigan School of Dentistry Ann Arbor Michigan USA

**Keywords:** bleeding on probing, bone loss, modified bleeding index, peri‐implant health, peri‐implantitis, supportive peri‐implant care

## Abstract

**Introduction:**

A study was made to assess the diagnostic and predictive value of bleeding on probing (BoP) and the modified Bleeding Index (mBI) for identifying progressive peri‐implant bone loss over a 24‐month period in patients enrolled in supportive peri‐implant care.

**Materials and Methods:**

A prospective cohort study was carried out of 59 patients with screw‐retained implant‐supported prostheses followed‐up on for 24 months. Clinical parameters, including BoP, mBI, probing depth and plaque index, were recorded at six sites per implant across five follow‐up visits. Progressive peri‐implant bone loss was defined as ≥ 0.5 mm of marginal bone loss as assessed radiographically. The longitudinal diagnostic performance of the bleeding indices was evaluated using sensitivity, specificity, positive predictive value (PPV), negative predictive value (NPV), and receiver operating characteristic (ROC) analyses.

**Results:**

After 24 months, 9 of 59 implants (15.3%) demonstrated progressive bone loss. Both BoP and mBI showed high sensitivity (88.9%–100%) but low specificity (0%–26%) for detecting bone loss. PPV was low (16%–18%), whereas NPV remained high (91%–100%). Recurrent low‐grade bleeding (mBI = 1) observed across multiple visits was more strongly associated with progressive bone loss than isolated episodes of severe bleeding. Consistent absence of bleeding was associated with peri‐implant stability.

**Conclusions:**

In patients receiving supportive peri‐implant care, the absence of bleeding is a reliable indicator of peri‐implant health. While bleeding assessed at a single timepoint has limited specificity, longitudinal patterns of mild bleeding are of greater value in predicting disease progression, highlighting the importance of regular monitoring and of standardized clinical assessment protocols. This prospective cohort study was not registered prior to participant recruitment and randomization.

## 1 Introduction

Peri‐implant mucositis is defined as inflammation confined to the peri‐implant soft tissues with no associated bone loss, and its effective management is the main preventive strategy against peri‐implantitis. If left untreated, mucositis may transition to peri‐implantitis, leading to progressive destruction of the supporting bone [1]. Because the treatment of peri‐implantitis shows variable and often limited long‐term success, early detection and continuous monitoring of the peri‐implant tissues are essential for maintaining implant health [2].

Among the clinical parameters used for peri‐implant monitoring, bleeding on probing (BoP) is traditionally considered the primary indicator of soft tissue inflammation [3]. In the natural dentition, BoP has been associated with an increased risk of attachment loss and a compromised tooth prognosis [4].

However, the diagnostic and prognostic accuracy of BoP remains limited. Systematic reviews indicate that while BoP effectively identifies inflammation, its ability to predict progressive bone loss is moderate. In a meta‐analysis including more than 17 000 implants, Yu et al. reported that 26.5% of BoP‐positive implants and 35.1% of BoP‐positive patients were diagnosed with peri‐implantitis [5]. Comparable figures were reported by Hashim et al. with approximately 24.1% of BoP‐positive implants exhibiting peri‐implantitis [6]. These data suggest that although BoP can detect inflammation, it has only moderate predictive value in differentiating between peri‐implant mucositis and peri‐implantitis. In this regard, the recent clinical practice guidelines of the European Federation of Periodontology emphasize that BoP must be interpreted in combination with probing depths and radiographic assessments [2]. Furthermore, the presence of BoP may be influenced by probing force, tissue phenotype, keratinized mucosa, and patient‐related factors such as smoking or systemic conditions. Despite the widespread use of BoP, evidence on the longitudinal relationship between this parameter and progressive bone loss remains limited [7].

Considering the ongoing debate regarding the diagnostic value of BoP, there is a clear need for prospective studies evaluating whether bleeding indices can reliably capture progressive tissue breakdown over time.

Thus, the aim of this prospective cohort study was to assess the diagnostic accuracy of BoP for detecting progressive radiographic bone loss over a 24‐month period in patients enrolled in a supportive peri‐implant care program. Understanding the predictive value of BoP over time is essential for refining diagnostic protocols, better identifying patients and implant sites at increased risk of deterioration, and optimizing individualized treatments and supportive care strategies. We hypothesized that longitudinal patterns of BoP and modified bleeding index (mBI) values during supportive peri‐implant care are predictive of progressive peri‐implant bone loss over time, and that the persistent absence of bleeding is associated with peri‐implant stability.

## 2 Materials and Methods

### 2.1 Ethical Issues

This study was conducted in accordance with the principles outlined in the Declaration of Helsinki (2013) and ethical guidelines for research with human subjects. The research protocol was approved by the Ethics Committee of the Universitat Internacional de Catalunya (UIC) (Spain) (Ref. PER‐ECL‐2020‐01) prior to participant recruitment. All participants provided written informed consent prior to enrollment.

### 2.2 Study Design

The present study was designed as an observational prospective cohort study with a 24‐month follow‐up period. No interventions were assigned for research purposes, and all participants received routine supportive peri‐implant care according to standard clinical practice. Patients were recruited consecutively from the Department of Periodontology at the UIC until the required sample size was reached. Reporting of the study followed the STROBE guidelines.

### 2.3 Study Population

Patients attending the Department of Periodontology for supportive periodontal and peri‐implant care were screened for eligibility. Inclusion criteria were adults aged 18 years or older with at least one screw‐retained implant‐supported prosthesis, presenting a minimum of 2 mm of keratinized mucosa at all evaluated sites. Both single and partial fixed implant prostheses were included. Included implants had been in function for at least 6 months prior to enrollment. After inclusion, all the participants followed the same standardized supportive care and follow‐up protocol.

Exclusion criteria included the presence of peri‐implantitis or previously treated peri‐implantitis, 9 or more periodontal pockets deeper than 5 mm, and a full‐mouth bleeding score > 25%. Implants with inadequate prosthesis design preventing insertion of an interproximal brush ≥ 0.4 mm at mesial and distal sites were excluded, as were cemented implant prostheses, current smokers or former smokers who quit within the past 10 years, patients taking anticoagulants, antiplatelet drugs, nonsteroidal antiinflammatory drugs, or systemic/local antibiotics within 2 weeks prior to assessment.

### 2.4 Definitions

Peri‐implant status was classified according to the classification of Periodontal and Peri‐implant diseases edited in 2017 and published in 2018 [3]:

- **Peri‐implant health:** No clinical signs of inflammation; no bleeding on probing; no suppuration; stable marginal bone levels; no increase in probing depth.
- **Peri‐implant mucositis:** Presence of bleeding on probing and/or suppuration, without radiographic evidence of progressive bone loss.
- **Peri‐implantitis:** Bleeding on probing and/or suppuration, increased probing depths, with radiographic evidence of progressive bone loss.

**Progressive bone loss** was defined as an increase of ≥ 0.5 mm in marginal bone loss at the end of the follow‐up period.

### 2.5 Examination

#### 2.5.1 Screening Examination

During the screening examination, the same calibrated investigator (GT) consecutively examined the patients and was responsible for enrolling them. The clinician reviewed with the patient the Information and Medication History Forms, and recorded the anthropometric, sociodemographic, and clinical data.

All patients underwent an oral pathology examination and full‐mouth manual probing to determine their periodontal and peri‐implant status. Finally, periapical radiographs (Dürr Dental AG, Bietigheim‐Bissingen, Germany) of all implants were taken using a paralleling cone technique and a film‐holder (7 mA, 60 kV/20 ms) in order to assess the marginal bone level prior to final recruitment, and to exclude patients with peri‐implantitis.

##### 2.5.1.1 Handling of Correlated Data

To avoid issues related to correlated observations, only one implant per patient was randomly selected for analysis. Therefore, each observation represents an independent unit, and no adjustments for within‐patient correlation were necessary.

For all patients meeting the inclusion criteria, one implant was randomly selected using the “RAND” function in Excel 2016 (Microsoft, Redmond, Washington, USA).

##### 2.5.1.2 Measurement Standardization

A silicone bite block (Elite HD +, Zhermack, Verona, Italy) was fabricated intraorally including the buccal and lingual surfaces of the implant included in the study, and of the two adjacent teeth. Furthermore, a silicone index (Elite HD +, Zhermack, Verona, Italy) was fabricated to standardize probing angulation, and 6 grooves were created in correspondence of implant crown with a parallel direction with respect to the longitudinal axis of the implant (mesiobuccal [mb], midbuccal [b], distobuccal [db], mesiolingual [ml], midlingual [l], and distolingual [dl]). These grooves allowed the exact reproducibility of both direction and parallel angulation of the probe during the following visits.

#### 2.5.2 Subsequent Visits

At each study visit (baseline, V1; 6 months, V2; 12 months, V3; 18 months, V4; 24 months, V5), a standardized parallel radiograph of the selected implant was taken. Subsequently, the following implant‐level parameters were recorded at 6 sites per implant using a controlled force periodontal probe PA_ON calibrated to a probing force of 0.20 N (Orange Dental, Aspachstr, Biberach, Germany).

- Modified Plaque Index (mPI, 0‐3) [8]
  ○ Score 0: No detection of plaque.
  ○ Score 1: Plaque only recognized by running a probe across the smooth marginal surface of the implant.
  ○ Score 2: Plaque visible to the naked eye.
  ○ Score 3: Abundance of soft matter.
- Modified Bleeding Index (mBI, 0–3) [8]
  ○ Score 0: No bleeding on probing.
  ○ Score 1: Isolated bleeding spot visible.
  ○ Score 2: Blood forming a confluent red line on the margin.
  ○ Score 3: Heavy or profuse bleeding.
- Suppuration on probing
- Probing pocket depth (PPDi): Expressed in millimeters as the distance between the peri‐implant mucosal margin and the bottom of the pocket reached with the pressure of the electronic probe
- Implant mucosal recession (MRi). Expressed in millimeters as the distance between the mucosal margin and the supragingivally located top of the abutment.
- Implant Mucosa Index (IMI, 0‐4) [9]
  ○ Score 0: No bleeding after probing
  ○ Score 1: Minimal, single‐point bleeding
  ○ Score 2: Moderate, multipoint bleeding
  ○ Score 3: Profuse, multipoint bleeding
  ○ Score 4: Suppuration
- Bleeding Time Index (BTI, 0‐4) [10]
  ○ Score 0: No gingival bleeding after the first and the second gingival stimulation
  ○ Score 1: Bleeding between 6 and 15 s after the second probing stimulation.
  ○ Score 2: Gingival bleeding occurs between 11 and 15 s after first probing, or within 5 s of the second probing.
  ○ Score 3: Gingival bleeding occurs within the first 10 s after first probing.
  ○ Score 4: Spontaneous gingival bleeding occurs prior to stimulation.
- Width of keratinized tissue (KT). Measured at three buccal and lingual sites. With the help of a manual periodontal probe (UNC15/PH6, HuFriedy, Rockwell St., Chicago, IL, USA), the buccal and lingual peri‐implant mucosa was gently pushed coronally from apical of the mucogingival junction to detect the distance (mm) between the mucosal margin and mucogingival junction

Bleeding indices (mBI, MRi, IMI, and BTI) were recorded at 6 peri‐implant sites and expressed as the maximum score observed per implant, reflecting the highest degree of bleeding severity.

### 2.6 Supportive Peri‐Implant Care Protocol

At the end of each visit, patients received an identical supportive periodontal and peri‐implant care protocol. At each scheduled visit, professional mechanical plaque removal was performed using ultrasonic instruments with metal tips and stainless‐steel curettes, followed by air‐polishing with low‐abrasive erythritol powder on implant surfaces. Polishing of the teeth was performed with a low‐abrasive fluoride‐containing paste. Individualized oral hygiene reinforcement was implemented. Supportive care procedures were performed for all participants at the end of each visit, irrespective of the clinical parameter values, following the completion of outcome assessment.

### 2.7 Study Variables

The primary outcome was radiographic peri‐implant bone loss, assessed on standardized periapical radiographs at baseline and at each follow‐up visit (V1, V2, V3, V4, V5).

The digital radiographs were transferred into software (ImageJ; NIH, Bethesda, MD, USA), and a comparison between the radiographs was made. Marginal bone loss was determined by taking linear measurements from the most mesial and distal aspect of the implant platform to the first bone‐implant contact, corresponding to the marginal bone level. The length and diameter of the implant and the dimension of the threads were used to quantify the radiographic measurements. Progressive bone loss was defined as an increase of ≥ 0.5 mm in marginal bone loss at the end of the follow‐up period.

Secondary outcomes included:

- Modified Plaque Index (mPI) [8]
- Modified Bleeding Index (mBI) [8]
- Suppuration on probing
- Probing pocket depth (PPDi)
- Implant mucosal recession (MRi)
- Implant Mucosal Index (IMI) [9]
- Bleeding Time Index (BTI) [10]

### 2.8 Sample Size Calculation

The required sample size was determined a priori to detect an area under the curve (AUC) of 0.75 versus the null AUC of 0.50, with *α* = 0.05 (two‐sided) and *β* = 0.20 (statistical power 80%). Assuming a 3:1 allocation between negative and positive outcome groups [11], the minimum number of analyzable study units (implants; one per patient) was 56. To account for an anticipated 15% attrition, the target enrollment figure was increased to 66.

### 2.9 Intra‐Examiner Reliability

The examiner (GT) was calibrated prior to the study by assessing intra‐examiner reliability for PPDi and mBI. Calibration was conducted in five patients diagnosed with peri‐implant mucositis who were not included in the study cohort, and measurements were repeated on two occasions separated 5 days apart.

Radiographic assessments were performed by a single calibrated examiner (BdT). Calibration was carried out using 10 implant radiographs not included in the study, which were re‐evaluated after 48 h. The intraclass correlation coefficient (ICC) values exceeded 0.80 for all measurements, indicating good intra‐examiner agreement.

### 2.10 Statistical Analysis

The diagnostic and predictive performance of the bleeding parameters was evaluated using both cross‐sectional (per visit) and longitudinal (cumulative) frameworks. Only participants who completed the 24‐month follow‐up period and had complete outcome data were included in the final analysis.

#### 2.10.1 Cross‐Sectional Analysis

At each follow‐up visit, the association between each clinical parameter (BoP, mBI, IMI, BTI) and progressive radiographic bone loss at 24 months (> 0.5 mm) was assessed. Sensitivity, specificity, positive predictive value (PPV) and negative predictive value (NPV) were calculated. Receiver operating characteristic (ROC) curves and AUC values were obtained for each parameter at each visit.

#### 2.10.2 Longitudinal Prediction (Primary Analytical Framework)

To evaluate the ability of the bleeding parameters to predict future bone loss, a cumulative visit‐level approach was adopted. For each implant and for each clinical parameter (BoP, mBI, IMI, BTI), the number of visits with a “positive” finding up to 24 months was counted. Diagnostic performance metrics were then calculated for cumulative thresholds (≥ 1/5, ≥ 2/5, ≥ 3/5, ≥ 4/5, and 5/5 positive visits).

For mBI, three monotonic visit‐level definitions of positivity were used:

1. mBI ≥ 1 (any bleeding)
2. mBI ≥ 2 (moderate or profuse bleeding)
3. mBI = 3 (profuse bleeding only)

ROC curves were generated for each threshold definition.

#### 2.10.3 Additional Variables

Other peri‐implant parameters (PPDi, mPI, IMI, BTI, KT, MRi) were summarized descriptively and reported stratified by bone‐loss outcome. Given the limited number of events (*n* = 9), no multivariate modeling was performed in accordance with statistical guidance. All analyses were conducted at implant level.

## 3 Results

### 3.1 Patient and Implant Characteristics

Fifty‐nine patients completed the 24‐month follow‐up period. Of the 7 patients lost to follow‐up, two continued treatment in other dental centers and did not return; one patient died; one developed severe illness preventing attendance to follow‐up; one patient started anticoagulation therapy; and two patients could not be contacted. The median age was 63.0 years (interquartile range [IQR] 58.0–71.8), with a balanced sex distribution (28 women, 31 men). Systemic medication was reported by 16/59 participants (27.1%). Most implants were located in posterior regions (mandible 42.4%, maxilla 37.3%). Time in function was ≥ 3 years for two‐thirds of the implants, and nearly all were internal‐connection fixtures (98.3%). According to diagnostic criteria, 8 implants were classified as presenting peri‐implant health, and 51 as presenting peri‐implant mucositis at baseline. No meaningful differences were observed between patients with and without progressive bone loss. Baseline characteristics of the patients and implants are presented in Table 1.

**TABLE 1:** Baseline characteristics of patients and implants.

Variable	Total (*N* = 59)	≤ 0.5 mm (*n* = 50)	> 0.5 mm (*n* = 9)	*p*
Age, median (P25–P75), years	63.0 (58.0–71.8)	63.0 (58.0–71.0)	64.0 (60.0–72.0)	0.651
Sex, *n* (%)				> 0.999
Female	28 (47.5)	24 (48.0)	4 (44.4)	
Male	31 (52.5)	26 (52.0)	5 (55.6)	
Systemic medication, *n* (%)				> 0.999
No	43 (72.9)	36 (72.0)	7 (77.8)	
Yes	16 (27.1)	14 (28.0)	2 (22.2)	
Implant location, *n* (%)				0.339
Mandibular Posterior	25 (42.4)	23 (46.0)	2 (22.2)	
Maxillary posterior	22 (37.3)	17 (34.0)	5 (55.6)	
Mandibular anterior	4 (6.8)	4 (8.0)	0 (0.0)	
Maxillary anterior	8 (13.6)	6 (12.0)	2 (22.2)	
Time from prosthetic loading, median (P25–P75), years	4.10 (2.70–5.80)	3.90 (2.47–5.70)	4.10 (3.80–8.70)	0.213
Implant length, median (P25–P75), mm	10.0 (10.0–11.5)	10.0 (10.0–11.0)	10.0 (8.0–12.0)	0.982
Implant diameter, median (P25–P75), mm	4.00 (3.55–4.20)	4.10 (3.52–4.20)	4.00 (4.00–4.10)	0.670
Previous bone augmentation, *n* (%)				0.670
No	46 (78.0)	38 (76.0)	8 (88.9)	
Yes	13 (22.0)	12 (24.0)	1 (11.1)	
Implant neck surface, *n* (%)				0.053
Smooth	43 (72.9)	39 (78.0)	4 (44.4)	
Rough	16 (27.1)	11 (22.0)	5 (55.6)	
Implant connection, *n* (%)				
Internal	58 (98.3)	49 (98.0)	9 (100)	
External	1 (1.7)	1 (2.0)	0 (0.0)	
Implant brand, *n* (%)				0.785
Straumann (all variants)	17 (28.8)	14 (28.0)	3 (33.3)	
Klockner (all variants)	18 (30.5)	14 (28.0)	4 (44.4)	
Astra Tech	7 (11.9)	7 (14.0)	0 (0.0)	
BioHorizons	6 (10.2)	5 (10.0)	1 (11.1)	
Other brands	11 (18.6)	10 (20.0)	1 (11.1)	

*Note:* Data are presented as median (P25–P75) or number (%). Progressive peri‐implant bone loss was defined as > 0.5 mm at 24 months.

### 3.2 Bone Loss Outcome

At 24 months, 9/59 implants (15.3%) exhibited progressive radiographic bone loss (> 0.5 mm), whereas 50/59 (84.7%) remained stable.

### 3.3 Cross‐Sectional Diagnostic Performance

Cross‐sectional diagnostic metrics, including sensitivity, specificity, PPV, NPV, and AUC, for all bleeding indices (BoP, mBI, IMI, BTI) are reported collectively in Table 2.

**TABLE 2:** Cross‐sectional diagnostic performance of bleeding indices for the detection of progressive peri‐implant bone loss.

Index	AUC (95% CI)	Sensitivity	Specificity	PPV	NPV
BoP	0.544–0.574	0.889–1.000	0.100–0.260	0.167–0.178	0.909–1.000
MSBI ≥ 1	0.370–0.611	0.889–1.000	0.100–0.280	0.167–0.182	0.917–1.000
MSBI ≥ 2	0.370–0.611	0.111–0.444	0.380–0.680	0.053–0.200	0.760–0.872
MSBI = 3	0.370–0.611	0.000–0.111	0.880–0.960	0.000–0.333	0.830–0.857
IMI ≥ 1	0.451–0.701	0.889–1.000	0.100–0.280	0.167–0.182	0.917–1.000
IMI ≥ 2	0.451–0.701	0.333–0.778	0.340–0.640	0.132–0.280	0.810–0.941
IMI ≥ 3	0.451–0.701	0.000–0.222	0.800–0.940	0.000–0.250	0.833–0.863
BTI ≥ 1	0.272–0.696	0.889–1.000	0.100–0.280	0.167–0.182	0.917–1.000
BTI ≥ 2	0.272–0.696	0.444–0.778	0.180–0.580	0.109–0.250	0.692–0.935
BTI ≥ 3	0.272–0.696	0.111–0.556	0.420–0.760	0.033–0.294	0.724–0.905
BTI ≥ 4	0.272–0.696	0.000–0.000	0.960–0.980	0.000–0.000	0.842–0.845

*Note:* Values are reported as ranges across visits #1–#5. Progressive peri‐implant bone loss was defined as > 0.5 mm.

Abbreviations: AUC = area under the receiver operating characteristic (ROC) curve; BoP = bleeding on probing (overall measure); BTI = bleeding time index (overall measure); CI = confidence interval; IMI = implant mucosal index (overall measure); MSBI = sulcular bleeding index (overall measure); NPV = negative predictive value; PPV = positive predictive value; Sensitivity = true‐positive rate; Specificity = true‐negative rate.

Across the five visits, BoP showed consistently high sensitivity (88.9%–100%), but very limited specificity (1%–26%). Consequently, PPV remained low (16.7%–17.8%), whereas NPV was uniformly high (91%–100%).

These findings indicate that BoP‐negative implants rarely developed bone loss, while BoP‐positive implants frequently constituted false positives. The ROC analysis yielded AUC values between 0.54 and 0.57 across visits.

A similar diagnostic pattern was observed for mBI, IMI, and BTI when low bleeding thresholds (≥ 1) were applied, with high sensitivity and NPV, but low specificity and PPV. When higher bleeding severity thresholds were considered (mBI ≥ 2 or = 3; IMI ≥ 2 or ≥ 3; BTI ≥ 2 to ≥ 4), sensitivity decreased while specificity increased, although positive predictive values remained low and AUC values indicated poor to marginal discriminative performance.

### 3.4 Longitudinal Diagnostic Performance (Primary Analysis)

Longitudinal diagnostic metrics, including sensitivity, specificity, PPV, NPV, and AUC, for mBI are reported collectively in Table 3 and for IMI, BTI in Tables S1 and S2, respectively.

**TABLE 3:** Diagnostic performance of longitudinal mBI according to threshold and number of positive visits for predicting progressive peri‐implant bone loss (> 0.5 mm).

mBI definition/cut‐off	AUC (95% CI)	Sensitivity	Specificity	PPV	NPV
mBI ≥ 1					
≥ 1 visit	0.604 (0.458–0.751)	1.000	0.060	0.161	1.000
≥ 2 visits	0.604 (0.458–0.751)	1.000	0.100	0.167	1.000
≥ 3 visits	0.604 (0.458–0.751)	1.000	0.160	0.176	1.000
≥ 4 visits	0.604 (0.458–0.751)	0.889	0.260	0.178	0.929
≥ 5 visits	0.604 (0.458–0.751)	0.778	0.400	0.189	0.909
mBI ≥ 2					
≥ 1 visit	0.403 (0.224–0.583)	0.667	0.320	0.150	0.842
≥ 2 visits	0.403 (0.224–0.583)	0.333	0.480	0.103	0.800
≥ 3 visits	0.403 (0.224–0.583)	0.111	0.580	0.045	0.784
≥ 4 visits	0.403 (0.224–0.583)	0.111	0.700	0.062	0.814
≥ 5 visits	0.403 (0.224–0.583)	0.111	0.780	0.083	0.830
mBI ≥ 3					
≥ 1 visit	0.471 (0.357–0.585)	0.111	0.840	0.111	0.840
≥ 2 visits	0.471 (0.357–0.585)	0.000	0.920	0.000	0.836
≥ 5 visits	0.471 (0.357–0.585)	0.000	0.980	0.000	0.845

*Note:* Progressive peri‐implant bone loss was defined as > 0.5 mm at 24 months. mBI was evaluated longitudinally as the number of follow‐up visits exceeding predefined thresholds (≥ 1, ≥ 2, ≥ 3). No cut‐offs at three or four visits were available for mBI ≥ 3, as no patients met these criteria.

#### 3.4.1 Bleeding on Probing (BoP)

Using cumulative thresholds (≥ 1 to 5 positive visits), BoP maintained perfect sensitivity (100%) for thresholds ≥ 1 to 3/5, but with very low specificity (6%–14%). PPV remained modest (16.1%–17.6%), while NPV was 100%.

With stricter cut‐off points (≥ 4–5/5), specificity improved modestly (24%–38%) and PPV increased slightly (maximum 18.4%), at the cost of reduced sensitivity (88.9%–77.8%), whereas NPV stayed high (92.9%–90.9%). The ROC analysis showed limited discriminative accuracy across visits (e.g., Visit 5: AUC 0.592; 95% CI 0.444–0.740). Overall, the repeated absence of BoP was a strong indicator of stability, whereas the persistence of BoP over time had limited predictive value.

#### 3.4.2 Modified Bleeding Index (mBI, Longitudinal)

##### 3.4.2.1 Primary Definition: mBI ≥ 1 (Any Bleeding)

Repeated mBI ≥ 1 across visits provided the best longitudinal discrimination among all mBI definitions, with an AUC of 0.716 (95% CI 0.531–0.900).

Implants with 0/5 positive visits had a very low risk of progression, whereas implants with repeated positivity, particularly ≥ 3/5 visits, showed a higher proportion of cases within the bone‐loss group.

##### 3.4.2.2 Sensitivity Analysis: mBI ≥ 2

Increasing the bleeding severity threshold reduced sensitivity but increased specificity across cumulative categories. The discriminative accuracy decreased 0.403 (95% CI 0.224–0.583), indicating that moderate or profuse bleeding was less predictive than “any bleeding” when assessed cumulatively.

##### 3.4.2.3 Exploratory Definition: mBI = 3

Profuse bleeding showed poor predictive value, with low sensitivity, increasing specificity with stricter cut‐off points, and an AUC of 0.471 (95% CI 0.357–0.585). This reflected the low frequency of severe bleeding events in the cohort.

#### 3.4.3 Additional Bleeding Indices (IMI and BTI)

Both IMI and BTI showed patterns similar to BoP: high sensitivity at lower thresholds but low specificity and limited PPV. Their AUCs ranged from 0.45 to 0.60, indicating marginal discriminative ability.

## 4 Discussion

The present prospective cohort study evaluated the diagnostic value of soft‐tissue inflammatory parameters for detecting progressive peri‐implant bone loss in patients enrolled in supportive peri‐implant care. Across all bleeding metrics, the absence of bleeding over multiple maintenance visits emerged as the most consistent indicator of peri‐implant stability, whereas the presence or repetition of bleeding, regardless of its clinical intensity, showed a limited ability to identify implants at risk of future bone loss.

At all five time points, both BoP and mBI showed very high sensitivity but low specificity. Consequently, many stable implants also presented bleeding, resulting in low positive predictive values. In contrast, the absence of bleeding consistently demonstrated a strong association with radiographic stability, reflected in high NPV across visits.

These findings align with classical periodontal literature evaluating periodontal attachment loss. Lang et al. reported a low predictive value (6%) but high NPV (98%) of BoP for attachment loss over 30 months in patients receiving supportive therapy [12]. Subsequent studies similarly observed minimal periodontal progression in patients with low full‐mouth bleeding scores (< 20%), whereas higher bleeding scores correlated with increased attachment loss [13]. In implant populations, prospective studies have confirmed high NPV (82%–97%) and low PPV (10%–19%) for BoP in predicting peri‐implant tissue loss [14].

Some studies, however, report contrasting results. A retrospective study found a modest relationship between BoP and peri‐implant bone loss after 1 year of function, with a mean bone loss of 0.55 mm per bleeding site, but no correlation at longer follow‐ups (5–15 years) [15]. Another study in patients receiving periodontal and peri‐implant supportive therapy reported a high PPV for BoP at implant sites when present in ≥ 50% of visits, possibly due to a higher threshold for defining progressive attachment loss (≥ 2.5 mm radiographically or 0.5 mm/year clinically) [16].

The variability in BoP predictive performance can be partly explained by methodological factors such as probing force, which significantly affects BoP outcomes. Gerber et al. demonstrated that increasing probing force from 0.15 to 0.25 N raised BoP by 14%, potentially leading to overdiagnosis [17]. Importantly, implants are inherently more susceptible to “false positive” bleeding compared with natural teeth, due to differences in peri‐implant connective tissue. As a result, bleeding may occur during probing as a consequence of minor mechanical trauma or probe angulation, instead of reflecting active inflammation. This biological vulnerability has led to recent refinements in the classification of peri‐implant diseases, now recognizing that implants may still be considered healthy despite the presence of a single bleeding site (mBI = 1) among six peri‐implant sites [2]. These updates reinforce the need for cautious interpretation of BoP findings and support the concept that bleeding absence is a more reliable indicator of peri‐implant health than bleeding presence alone.

It should be noted that the present study was designed according to the 2018 classification system [3], which was applicable at the time of study initiation, and defined any BoP as peri‐implant mucositis. Consequently, bleeding indices were recorded using the maximum mBI score observed across the 6 sites per implant to capture bleeding severity, and the present analysis does not distinguish between isolated single‐site bleeding and bleeding affecting multiple sites. While some implants in our analysis could thus be classified as healthy under the updated criteria, this does not affect the key finding: the absence of bleeding remains a robust indicator of peri‐implant stability.

A notable observation in our study was the low overall percentage of implants experiencing marginal bone loss ≥ 0.5 mm over 24 months (15.3%), supporting the preventive role of structured supportive peri‐implant therapy [18]. Additionally, the frequency of bleeding over time appeared more informative than its severity. In effect, recurrent low‐grade inflammation (mBI = 1) showed stronger associations with progressive bone loss than occasional severe bleeding. This suggests that chronic, subtle inflammatory challenges may better reflect tissue vulnerability than sporadic intense episodes.

Strengths of this study include its prospective design, standardized supportive peri‐implant therapy with biannual visits, the use of multiple bleeding indices, controlled probing forces, and standardized periapical radiographs minimizing measurement errors. Limitations include the exclusion of smokers (to avoid nicotine‐related effects on BoP), and the 24‐month follow‐up period, which may reduce statistical power and limit the generalizability of findings.

From a clinical perspective, the findings emphasize the importance of consistent supportive peri‐implant therapy. Clinicians should monitor peri‐implant tissues not only through BoP but in combination with probing depth measurements and radiographic assessments in order to detect early changes.

Future investigations should prioritize longer follow‐up periods (> 5 years) and larger cohorts to strengthen the validity and generalizability of these findings. Importantly, future study designs should refine bleeding categorization by avoiding the interpretation of a single bleeding spot (mBI = 1 at one of six sites) as a stand‐alone indicator of inflammation, and instead emphasize longitudinal patterns and site distribution. Further standardization of probing techniques, particularly regarding probing force, together with the integration of adjunctive biomarkers, may enhance predictive accuracy. In addition, the evaluation of systemic and patient‐related risk factors could help determine whether bleeding indices exhibit different predictive values across distinct risk profiles.

## 5 Conclusions

In patients receiving supportive peri‐implant care, the absence of bleeding is a reliable indicator of peri‐implant health. While bleeding assessed at a single timepoint exhibits limited specificity, longitudinal patterns of mild bleeding offer improved value for predicting disease progression, highlighting the importance of regular monitoring and standardized clinical assessment protocols.

## Author Contributions

A.M. and B.d.T. contributed to conceptualization of the study. The study design was developed by B.d.T., A.M., and E.A. Data collection and field work were performed by G.T. and F.d.L. Data analyses were conducted by G.T., B.d.T., and E.A. The manuscript was drafted by F.d.L., B.d.T., and E.A. Critical review of the manuscript for important intellectual content was carried out by A.M., J.N., and C.V. All authors reviewed and approved the final version of the manuscript.

## Funding

The authors have nothing to report.

## Conflicts of Interest

The authors declare no conflicts of interest.

## Supporting information


**Table S1:** Diagnostic performance of longitudinal IMI according to threshold and number of positive visits for predicting progressive peri‐implant bone loss (> 0.5 mm).

**Table S2:** Diagnostic performance of longitudinal BTI according to threshold and number of positive visits for predicting progressive peri‐implant bone loss (> 0.5 mm).

## Data Availability

The data that support the findings of this study are available on request from the corresponding author. The data are not publicly available due to privacy or ethical restrictions.
