# Supplementary material for: Bleeding on Probing as a Predictor of Peri‐Implant Bone Loss During Supportive Care: A Prospective Cohort Study
Source: Clin Implant Dent Relat Res. 2026 Mar 24;28(2):e70138. doi: 10.1111/cid.70138 (PMC13010781; doi:10.1111/cid.70138)
Supplement: Supplementary file 1 — Table S1: Diagnostic performance of longitudinal IMI according to threshold and number of positive visits for predicting progressive peri‐implant bone loss (> 0.5 mm). [file CID-28-0-s001.docx]

# Table S1. Diagnostic performance of longitudinal IMI according to threshold and number of positive visits for predicting progressive peri-implant bone loss (>0.5 mm)

| **IMI definition / cut-off** | **AUC (95% CI)** | **Sensitivity** | **Specificity** | **PPV** | **NPV** |  |
| --- | --- | --- | --- | --- | --- | --- |
| **IMI ≥ 1** |  |  |  |  |  |  |
| *≥1 visit* | 0.604 (0.458–0.751) | 1.000 | 0.060 | 0.161 | 1.000 |  |
| *≥2 visits* | 0.604 (0.458–0.751) | 1.000 | 0.100 | 0.167 | 1.000 |  |
| *≥3 visits* | 0.604 (0.458–0.751) | 1.000 | 0.160 | 0.176 | 1.000 |  |
| *≥4 visits* | 0.604 (0.458–0.751) | 0.889 | 0.260 | 0.178 | 0.929 |  |
| *≥5 visits* | 0.604 (0.458–0.751) | 0.778 | 0.400 | 0.189 | 0.909 |  |
| **IMI ≥ 2** |  |  |  |  |  |  |
| *≥1 visit* | 0.572 (0.373–0.771) | 1.000 | 0.260 | 0.196 | 1.000 |  |
| *≥2 visits* | 0.572 (0.373–0.771) | 0.556 | 0.380 | 0.139 | 0.826 |  |
| *≥3 visits* | 0.572 (0.373–0.771) | 0.444 | 0.520 | 0.143 | 0.839 |  |
| *≥4 visits* | 0.572 (0.373–0.771) | 0.444 | 0.720 | 0.222 | 0.878 |  |
| *≥5 visits* | 0.572 (0.373–0.771) | 0.333 | 0.780 | 0.214 | 0.867 |  |
| **IMI ≥ 3** |  |  |  |  |  |  |
| *≥1 visit* | 0.477 (0.312–0.642) | 0.222 | 0.720 | 0.125 | 0.837 |  |
| *≥2 visits* | 0.477 (0.312–0.642) | 0.111 | 0.900 | 0.167 | 0.849 |  |
| *≥3 visits* | 0.477 (0.312–0.642) | 0.111 | 0.960 | 0.333 | 0.857 |  |
| *≥5 visits* | 0.477 (0.312–0.642) | 0.000 | 0.960 | 0.000 | 0.842 |  |

Footnotes: Progressive peri-implant bone loss was defined as >0.5 mm at 24 months. IMI was evaluated longitudinally as the number of follow-up visits exceeding predefined thresholds.
