# Supplementary material for: Bleeding on Probing as a Predictor of Peri‐Implant Bone Loss During Supportive Care: A Prospective Cohort Study
Source: Clin Implant Dent Relat Res. 2026 Mar 24;28(2):e70138. doi: 10.1111/cid.70138 (PMC13010781; doi:10.1111/cid.70138)
Supplement: Supplementary file 2 — Table S2: Diagnostic performance of longitudinal BTI according to threshold and number of positive visits for predicting progressive peri‐implant bone loss (> 0.5 mm). [file CID-28-0-s002.docx]

# Table S2. Diagnostic performance of longitudinal BTI according to threshold and number of positive visits for predicting progressive peri-implant bone loss (>0.5 mm)

| **IMI definition / cut-off** | **AUC (95% CI)** | **Sensitivity** | **Specificity** | **PPV** | **NPV** |
| --- | --- | --- | --- | --- | --- |
| **BTI ≥ 1** |  |  |  |  |  |
| *≥1 visit* | 0.604 (0.458–0.751) | 1.000 | 0.060 | 0.161 | 1.000 |
| *≥2 visits* | 0.604 (0.458–0.751) | 1.000 | 0.100 | 0.167 | 1.000 |
| *≥3 visits* | 0.604 (0.458–0.751) | 1.000 | 0.160 | 0.176 | 1.000 |
| *≥4 visits* | 0.604 (0.458–0.751) | 0.889 | 0.260 | 0.178 | 0.929 |
| *≥5 visits* | 0.604 (0.458–0.751) | 0.778 | 0.400 | 0.189 | 0.909 |
| **BTI ≥ 2** |  |  |  |  |  |
| *≥1 visit* | 0.476 (0.264–0.687) | 1.000 | 0.140 | 0.173 | 1.000 |
| *≥2 visits* | 0.476 (0.264–0.687) | 0.556 | 0.240 | 0.116 | 0.750 |
| *≥3 visits* | 0.476 (0.264–0.687) | 0.556 | 0.360 | 0.135 | 0.818 |
| *≥4 visits* | 0.476 (0.264–0.687) | 0.444 | 0.520 | 0.143 | 0.839 |
| *≥5 visits* | 0.476 (0.264–0.687) | 0.333 | 0.660 | 0.150 | 0.846 |
| **BTI ≥ 3** |  |  |  |  |  |
| *≥1 visit* | 0.450 (0.252–0.648) | 0.667 | 0.340 | 0.154 | 0.850 |
| *≥2 visits* | 0.450 (0.252–0.648) | 0.333 | 0.480 | 0.103 | 0.800 |
| *≥3 visits* | 0.450 (0.252–0.648) | 0.222 | 0.700 | 0.118 | 0.833 |
| *≥4 visits* | 0.450 (0.252–0.648) | 0.222 | 0.820 | 0.182 | 0.854 |
| *≥5 visits* | 0.450 (0.252–0.648) | 0.000 | 0.900 | 0.000 | 0.833 |
| **BTI ≥ 4** |  |  |  |  |  |
| *≥1 visit* | 0.480 (0.453–0.507) | 0.000 | 0.960 | 0.000 | 0.842 |
| *≥5 visits* | 0.480 (0.453–0.507) | 0.000 | 0.980 | 0.000 | 0.845 |

Progressive peri-implant bone loss was defined as >0.5 mm at 24 months. BTI was evaluated longitudinally as the number of follow-up visits exceeding predefined thresholds.
